# Inflammation Activation Contributes to Adipokine Imbalance in Patients with Acute Coronary Syndrome

**DOI:** 10.1371/journal.pone.0151916

**Published:** 2016-03-17

**Authors:** Rong Li, Lu-zhu Chen, Shui-ping Zhao, Xian-sheng Huang

**Affiliations:** 1 Department of Stomatology, The Second Xiangya Hospital, Central South University, Changsha, Hunan, P.R. China; 2 Department of Cardiology, The Second Xiangya Hospital, Central South University, Changsha, Hunan, P.R. China; Albert Einstein College of Medicine, UNITED STATES

## Abstract

Inflammation can be activated as a defensive response by the attack of acute coronary syndrome (ACS) for ischemic tissue injury. The aim of the present study was to investigate the impact of ACS-activated inflammation on adipokine imbalance and the effects of statins on the crosstalk between inflammation and adipokine imbalance during ACS. In this study, 586 subjects were categorized into: (1) control group; (2) SA (stable angina) group; and (3) ACS group. Circulating levels of hs-CRP, adiponectin and resistin were measured by ELISA. Furthermore, forty C57BL/6 mice were randomized into: sham, AMI, low-statin (atorvastatin, 2 mg/kg/day) and high-statin (atorvastatin, 20 mg/kg/day) group. After 3 weeks, AMI models were established by surgical coronary artery ligation. Circulating levels and adipose expressions of adiponectin and resistin were assessed in animals. Besides, we investigate the effects of atorvastatin on ox-LDL-induced adipokine imbalance in vitro. As a result, we found that ACS patients had higher hs-CRP and resistin levels and lower adiponectin levels. Our correlation analysis demonstrated hs-CRP concentrations were positively correlated with resistin but negatively with adiponectin levels in humans. Our animal findings indicated higher circulating hs-CRP and resistin levels and lower adiponectin levels in AMI mice. Atorvastatin pre-treatment dose-dependently decreased hs-CRP and resistin levels but increased adiponectin levels in mice. The consistent findings were observed about the adipose expressions of resistin and adiponectin in mice. In study *in vitro*, ox-LDL increased cellular resistin expressions and otherwise for adiponectin expressions, which dose-dependently reversed by the addition of atorvastatin. Therefore, our study indicates that the ACS attack activates inflammation leading to adipokine imbalance that can be ameliorated by anti-inflammation of atorvastatin.

## Introduction

Atherosclerosis is acknowledged as an inflammatory disease and inflammation plays an important role in the development of atherosclerotic cardiovascular diseases. Besides involving in the formation of atherosclerotic plaque, inflammation is believed to result in the rupture of plaque that consequently develops into a setting of acute ischemic cardiovascular diseases, i.e. acute coronary syndrome (ACS) [1]. However, on the other hand, ACS represents an acute heart attack that would activate systemic inflammation as a defensive response for this ischemic tissue injury. During ACS-activated inflammation, these patients could undergo homeostatic imbalance that is likely to disturb the production and secretion of various cytokines. Recently, inflammation has been documented as a key participant in homeostatic imbalance of adipokines [2]. Nevertheless, to date, the impact of ACS-activated inflammation on adipokines remains unknown in ACS patients.

Adipose tissue is traditionally considered as a storage organ of surplus fat and energy. However, adipose tissue has been recently demonstrated as an endocrine organ that releases a variety of adipokines. In terms of the role in atherogenesis, adipokines can be divided into anti-atherogenic (such as adiponectin) and proatherogenic adipokines (such as resistin and leptin) [3–5]. Interestingly, inflammation and inflammation-associated diseases (such as diabetes and obesity) predispose adipose tissue into a dysfunctional endocrinal organ. As a result, it leads to proatherogenic adipokine imbalance with higher adiponectin levels as well as lower resistin and leptin levels [6]. Obviously, the adipokine imbalance induced by inflammation would likely increase the risk of atherosclerosis. Of note, ACS-activated inflammation as aforementioned should be categorized as an acute inflammation rather than a chronic inflammation (unlike the chronic inflammation in patients with diabetes, obesity, or even stable atherosclerotic cardiovascular diseases [1]). As far as we know, it is still uncertain whether the acute attack of ACS events would contribute to adipokine imbalance in these population. Besides, it is interesting in this study to identify the role of acute inflammation (i.e. ACS-activated inflammation) in bridging the ACS attack and adipokine imbalance.

Statins are recommended as a keystone medication for atherosclerotic cardiovascular disease. Beyond lipid-modulation effects, other anti-atherosclerotic effects of statins are also implicated in cardiovascular protection that are widely acknowledged as the pleiotropic effects of statins [7]. Among statin pleiotropic effects, anti-inflammatory property of this agent has been well-documented as a crucial contributor to prevent atherosclerotic cardiovascular events [8]. The administration of statins in the early period of ACS has been shown to reduce the levels of circulating inflammatory markers in these patients [9]. Therefore, the effects from statin anti-inflammatory property on ACS-induced adipokine imbalance was also to be investigated in this study.

## Materials and Methods

### Human study

#### Subjects

In this study, 586 subjects were recruited from the Second Xiangya Hospital, Central South University (Hunan, China) and categorized into: (1) control group (n = 190); (2) SA (stable angina) group (n = 192); and (3) ACS group (n = 204), including patients with unstable angina (UA) (n = 98) and acute myocardial infarction (AMI) (n = 106), who presented within 24 hours after the onset of symptoms. Exclusion criteria were: (1) severe hepatic or/and renal diseases; (2) severe heart failure; (3) other diseases, including diabetes mellitus, obesity, acute or recent (<2 months) infection, immunologic disorders, thyroid diseases, recent major trauma, and cancer; and (4) recently treatment with lipid-lowering, anti-inflammatory or immunosuppressive agents. The study protocol and consent procedure were approved by Ethics Committee of The Second Xiangya Hospital of Central South University. Written informed consents were obtained from all subjects participated in this study. All data were re-numbered and analyzed anonymously.

#### Laboratory measurements

Blood samples were obtained after an overnight fast and stored at −80°C until analysis. Serum levels of adiponectin (Linco Research) and resistin (Linco Research) were measured by ELISA kits according to instructions. The intra- and inter-assay coefficients of variation were < 5% and < 10%, respectively.

### Animal experiment

Experiments were carried out in accordance with a protocol (No. XY-2011-S168) approved by the Institutional Animal Care and Use Committee of Animal Experiment Department of Central South University. Adequate measures were taken to minimize pain or discomfort to experimental animals. Forty 10-week-old male C57BL/6 mice (Slac, Shanghai, China) were housed in an accredited institute facility in accordance with the institutional animal care policies, and were randomized into four groups (n = 10 each group): sham (a sham operation group, normal saline by oral gavage for 3 weeks), AMI (normal saline by oral gavage for 3 weeks), low-statin (atorvastatin, 2 mg/kg/day by oral gavage for 3 weeks, Pfizer) and high-statin (atorvastatin, 20 mg/kg/day for 3 weeks, Pfizer) groups. After 3 weeks, AMI models (all mice in AMI, low-statin and high-statin groups, rather than in sham group) were established by surgical ligation of the left coronary artery as described previously [10]. Prior to surgery, mice were anesthetized with isoflurane inhalation (4% induction followed by 1–2.5% maintenance). Subsequent to induction of anaesthesia, mice were orally intubated with polyethylene-60 (PE-60) tubing, connected to a mouse ventilator (MiniVent Type 845, Hugo-Sachs Elektronik) set at a tidal volume of 240 μL and a rate of 110 breaths per minute, and supplemented with oxygen. Body temperature was maintained at 37°C. A median sternotomy was performed, and the proximal left coronary artery (LAD) was visualized and ligated with 7–0 silk suture mounted on a tapered needle (BV-1, Ethicon). After 30min of ischemia, the prolene suture was cut and LAD blood flow restored. At 24 hours after AMI, all mice were euthanized and blood samples were collected and stored at −80°C until analysis. Serum levels of high sensitivity C-reactive protein (hs-CRP) (Abcam), adiponectin (Linco Research) and resistin (Linco Research) were assessed with ELISA kits. Fat pads were pooled from epididymal and retroperitoneal region, and stored at −80°C for Western blot analysis of adiponectin and resistin.

### Cell experiment

Mouse 3T3-L1 preadipocytes were obtained from American Type Culture Collection (Rockville, MD) and cultured in Dulbecco’s modified Eagle’s medium (DMEM, Cellgro Mediatech, Inc. Manassas, VA) containing 10% fetal bovine serum (FBS, Etobicoke, ON, Canada) until confluent, and were then seeded into 96-well plates in DMEM with 10% FBS, 100 U/mL penicillin and 100 μg/mL streptomycin in a humidified 5% CO_2_ incubator at 37°C. Subsequently, the cells were differentiated into mature adipocytes as described previously [11]. Differentiated 3T3-L1 adipocytes were starved in serum-free DMEM for 1 hour before stimulation. In this in vivo study, each group was assigned with 6 culture plates and each cell experiment had been performed in 3 repetitions.

Since oxidized low-density lipoproteins (ox-LDL) is a crucial proinflammatory factor during atherogenesis [12], it was been utilized as a stimulator to induce cellular inflammation in our study. All chemicals (including ox-LDL and atorvastatin) were dissolved in dimethyl sulphoxide (DMSO) and the final concentration of DMSO in media was maintained at 0.1% (v/v).

All mature adipocytes were divided into: (1) control group; (2) ox-LDL group, with 20 μg/ml ox-LDL; (3) low-statin group, with 20 μg/ml ox-LDL and a low dose (0.1mol/L) of atorvastatin; (4) high-statin group, with 20 μg/ml ox-LDL and a high dose (1mol/L) of atorvastatin. After 24 hour incubation, the expressions of adiponectin and resistin in cells were measured by Western blot analysis.

Since human resistin, unlike rodent resistin, is predominantly expressed in macrophages [13], human macrophages were used for testifying our hypothesis. Monocytes were isolated from human peripheral blood obtained from healthy volunteers and differentiated into macrophages, that were divided into: (1) control group; (2) ox-LDL group, with 20 μg/ml ox-LDL; (3) low-statin group, with 20 μg/ml ox-LDL and a low dose (0.1mol/L) of atorvastatin; (4) high-statin group, with 20 μg/ml ox-LDL and a high dose (1mol/L) of atorvastatin. After 24 hour incubation, the expressions of resistin in cells were determined by Western blot analysis.

### Western blot analysis

Equal amount (50 μg) of protein for each sample was loaded and separated on a 10% SDS-PAGE. After electrophoretic separation, the proteins were transferred onto PVDF membranes. According to each target protein, the membrane was incubated with mouse monoclonal antibody to adiponectin (mouse monoclonal antibody, ab22554, Abcam) or rabbit polyclonal antibody to resistin (ab119501, Abcam) or mouse monoclonal antibody to resistin (ab136877, Abcam), at 4°C for overnight. After incubation with horseradish peroxidase (HRP)-conjugated secondary antibody, immunoreactive protein bands were visualized using the enhanced chemiluminescence detection substrate photoreactive X-ray films. Densitometry quantification of protein band intensity was performed using the TINA software (Raytest, Straubenhardt, Germany). The expressions of each target protein were present as fold of the loading control, β-actin.

### Statistical analysis

All the statistical calculations were performed with SPSS 15.0 statistical software package (SPSS Inc). Data are presented as mean (±SD) or median [25th–75th percentiles] for continuous variables and as proportions for categorical variables. When two groups were to be compared, the significance was evaluated by unpaired Student t-test; when multiple groups were to be compared, the significance was evaluated by one-way ANOVA followed by the test of Student–Newman–Keuls. Partial Pearson correlation coefficients were used to establish the relationship between two variables, with adjustment for other potential confounders. Because serum hs-CRP levels have non-Gaussian distribution, the original hs-CRP values were transformed logarithmically, and correlation analyses between hs-CRP and adipokines (adiponectin and resistin) levels were also carried out across the tertiles of hs-CRP. Results were considered statistically significant at two-sided P < 0.05.

## Results

### Characteristics of subjects

The main characteristics of subjects are summarized in Table 1. As compared with controls, all patients with coronary heart disease (including SA and ACS) have higher levels of hs-CRP and resistin, but lower levels of adiponectin. Furthermore, the elevation of hs-CRP and resistin as well as the reduction of adiponectin were more significant in ACS patients than SA patients.

**Table 1:** Clinical and biochemical characteristics in subjects.

	Control	SA	ACS
Number	190	192	204
Gender (male/female)	92/98	95/97	104/100
Age (years)	55.8 ± 10.8	56.3 ± 11.5	56.7 ± 12.9
Smoking (current)	8 (20%)	20 (50%) ^a^	28 (70%) ^a^^,^^b^
BMI (kg/m^2^)	22.8 ± 3.2	23.1 ± 3.6	23.4 ± 3.4
Systolic blood pressure (mmHg)	123 ± 10	148 ± 15 ^a^	152 ± 17 ^a^
Diastolic blood pressure (mmHg)	62 ± 7	75 ± 10 ^a^	76 ± 12 ^a^
Free blood glucose (mmol/L)	5.8 ± 1.2	6.0 ± 1.3	8.5 ± 1.5 ^a^^,^^b^
HbA1c (%)	4.7 ± 0.6	4.8 ± 0.8	4.9 ± 0.7
Triglycerides (mmol/L)	1.1 ± 0.4	2.3 ± 0.5 ^a^	3.6 ± 0.7 ^a^^,^^b^
Total cholesterol (mmol/L)	4.0 ± 0.6	5.6 ± 1.3 ^a^	5.8 ± 1.2 ^a^
HDL cholesterol (mmol/L)	1.6 ± 0.7	1.0 ± 0.4 ^a^	0.7 ± 0.4 ^a^
LDL cholesterol (mmol/L)	2.2 ± 0.5	3.6 ± 0.7 ^a^	3.5 ± 0.8 ^a^
hs-CRP (mg/L)	1.2 ± 0.8	7.9 ± 5.6 ^a^	20.3 ± 8.1 ^a^^,^^b^
Resistin (ng/ml)	9.0 ± 3.2	17.4 ± 6.9 ^a^	29.0 ± 8.6 ^a^^,^^b^
Adiponectin (μg/ml)	10.5 ± 1.2	6.7 ± 1.8 ^a^	3.5 ± 2.3 ^a^^,^^b^

SA, stable angina; ACS, acute coronary syndrome; BMI, body mass index; HbA1c, haemoglobin A1c; HDL, high density lipoprotein; LDL, low density lipoprotein; hs-CRP, high-sensitivity C-reactive protein.

^a^ Compared with control group, P<0.05;

^b^ Compared with SA group, P<0.05.

### Relationship between hs-CRP and adipokines in subjects

We carried out a correlation analysis between adipokine levels and hs-CRP levels in all subjects (Table 2). Because serum hs-CRP levels have non-Gaussian distribution, the original hs-CRP values were transformed logarithmically, and correlation analyses between hs-CRP and adipokines (adiponectin and resistin) levels were performed across the tertiles of hs-CRP. As shown in Table 2, within the low, middle, and high tertiles of hs-CRP concentrations in all human participants, positive correlations were observed between hs-CRP and resistin levels (all P <0.01), whereas negative correlations were indicated between hs-CRP and adiponectin levels (all P <0.05). Consistently, after pooling all data in each group (including control, SA and ACS group, respectively), the similar relationship was also demonstrated between hs-CRP levels and the two adipokine levels.

**Table 2:** Correlations between adipokines and hs-CRP within tertiles of hs-CRP in all subjects.

	Resistin		Adiponectin	
hs-CRP (n)	r	P	r	P
**Control**				
All (127)	0.56	<0.01	−0.52	<0.01
Low (38)	0.52	<0.01	−0.48	<0.05
Middle (45)	0.60	<0.01	−0.56	<0.01
High (37)	0.64	<0.001	−0.53	<0.01
**SA**				
All (128)	0.63	<0.001	−0.56	<0.01
Low (37)	0.57	<0.01	−0.54	<0.01
Middle (47)	0.66	<0.001	−0.59	<0.001
High (44)	0.68	<0.001	−0.62	<0.001
**ACS**				
All (131)	0.67	<0.001	−0.56	<0.01
Low (42)	0.62	<0.001	−0.54	<0.01
Middle (48)	0.69	<0.001	−0.59	<0.001
High (40)	0.71	<0.001	−0.62	<0.001

hs-CRP, high-sensitivity C-reactive protein; SA, stable angina; ACS, acute coronary syndrome; r, Pearson correlation coefficient; P, P value. Low, Middle, High refer to the low, middle, and high tertiles of logarithmically-transformed hs-CRP levels.

### Statin on inflammation and adipokines in mice

As compared with sham group, all AMI mice (including mice in AMI and statin groups) have higher hs-CRP and resistin levels but lower adiponectin levels. Notably, the levels of hs-CRP and resistin were higher in mice treated with low-dose atorvastatin than those with high-dose atorvastatin. It indicated that atorvastatin dose-dependently attenuated the elevation of hs-CRP and resistin in AMI mice. However, opposite changes were observed about adiponectin levels in AMI mice with atorvastatin treatment (Table 3). Additionally, a correlation analysis was carried out between hs-CRP and adipokines (including resistin and adiponectin) levels in all animals. Similar findings as those in human study were observed in mice, i.e. hs-CRP levels in mice were positively correlated with resistin but negatively with adiponectin (Table 4).

**Table 3:** Serum levels of hs-CRP and adipokines in mice.

	Sham	AMI	Low-statin	High-statin
Number	10	10	10	10
hs-CRP (ng/ml)	10.6 ± 3.2	95.2 ± 10.6 ^a^	52.0 ± 6.2 ^a^^,^^b^	30.6 ± 4.5 ^a^^,^^b^^,^^c^
Resistin (ng/ml)	15.2 ± 2.5	57.2 ± 4.6 ^a^	35.2 ± 3.8 ^a^^,^^b^	28.6 ± 4.2 ^a^^,^^b^^,^^c^
Adiponectin (μg/ml)	7.6 ± 1.3	3.0 ± 0.9 ^a^	4.5 ± 0.6 ^a^^,^^b^	5.2 ± 0.8 ^a^^,^^b^^,^^c^

^a^ Compared with sham group, P<0.05;

^b^ Compared with AMI group, P<0.05;

^c^ Compared with low-statin group, P<0.05.

**Table 4:** Correlations between hs-CRP and adipokines in mice.

	Resistin		Adiponectin	
hs-CRP (n)	r	P	r	P
Sham (10)	0.42	<0.05	−0.40	<0.05
AMI (10)	0.53	<0.01	−0.50	<0.01
Low-statin (10)	0.48	<0.05	−0.43	<0.05
High-statin (10)	0.47	<0.05	−0.46	<0.05
All (40)	0.49	<0.05	−0.45	<0.05

hs-CRP, high-sensitivity C-reactive protein; Sham, Sham group; AMI, AMI group; Low-statin, low-statin (atorvastatin, 2 mg/kg/day) group; High-statin, high-statin (atorvastatin, 20 mg/kg/) group; r, Pearson correlation coefficient; P, P value.

**Fig 1:**
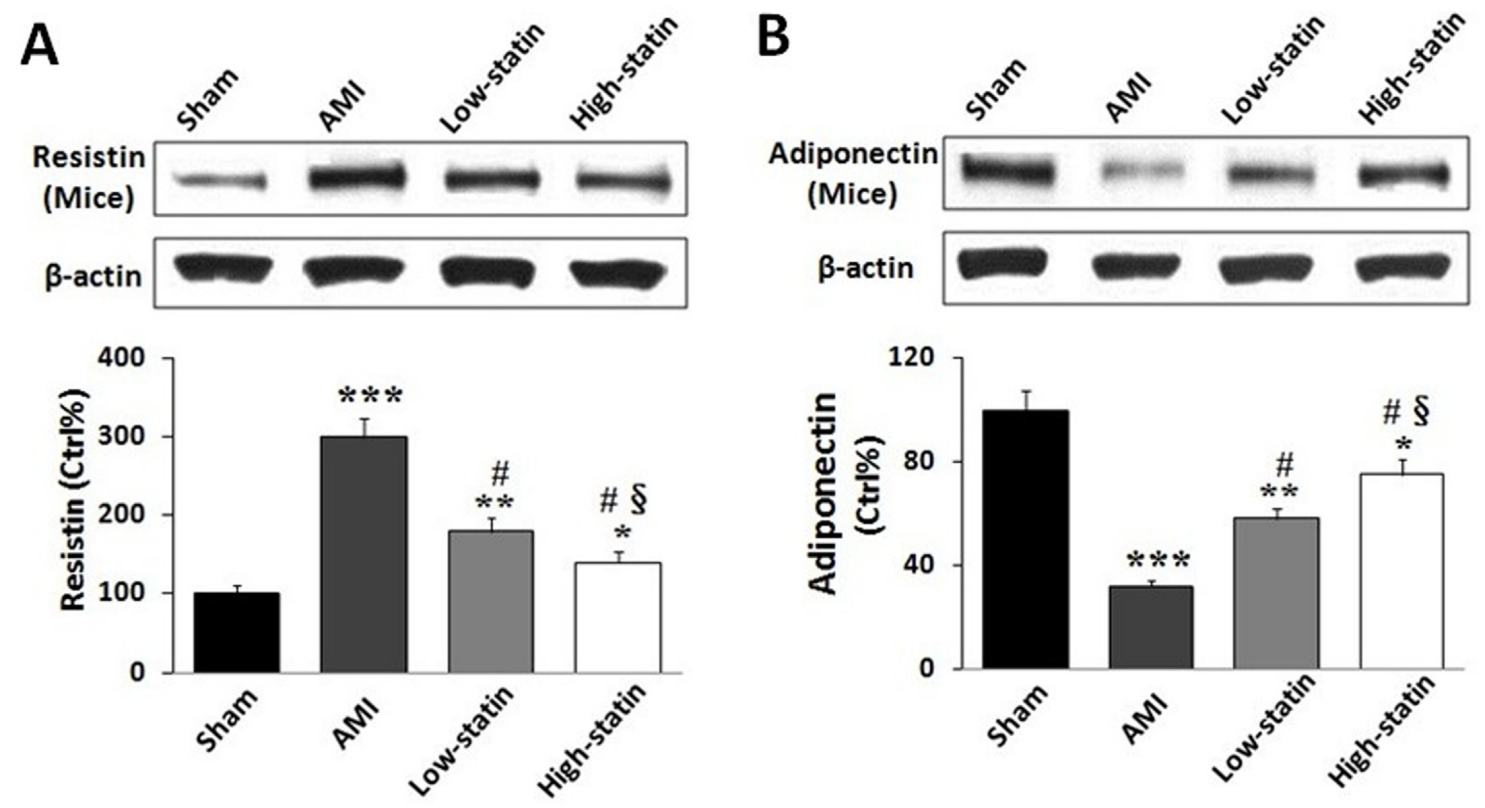
Effects of atorvastatin on resistin and adiponectin in AMI mice. (A) As compared with sham group, resistin expressions in adipose tissue were increased in all AMI mice. However, pre-treatment of atorvastatin dose-dependently attenuated AMI-induced up-regulation of resistin in mice. (B) Adiponectin expressions were markedly lower in all AMI mice than those in sham group. In contrast, administration of atorvastatin dose-dependently increased AMI-induced down- regulation of adiponectin in mice. *P < 0.05, **P < 0.01, ***P < 0.001 vs. control; # P < 0.01 vs. AMI; § P < 0.05 vs. low-statin group.

Furthermore, the expressions of resistin and adiponectin in adipose tissue were measured in mice by Western blot analysis. As shown in Fig 1, resistin expressions were significantly up-regulated in all AMI mice than those of sham group. However, atorvastatin pretreatment dose-dependently inhibited the expressions of resistin (Fig 1A). In contrast, adiponectin expressions were observed a significant reduction in all AMI mice than sham group. However, pretreatment of atorvastatin dose-dependently increased the expressions of adiponectin in AMI mice (Fig 1B).

### Statin on adipokines in cells treated with ox-LDL

**Fig 2:**
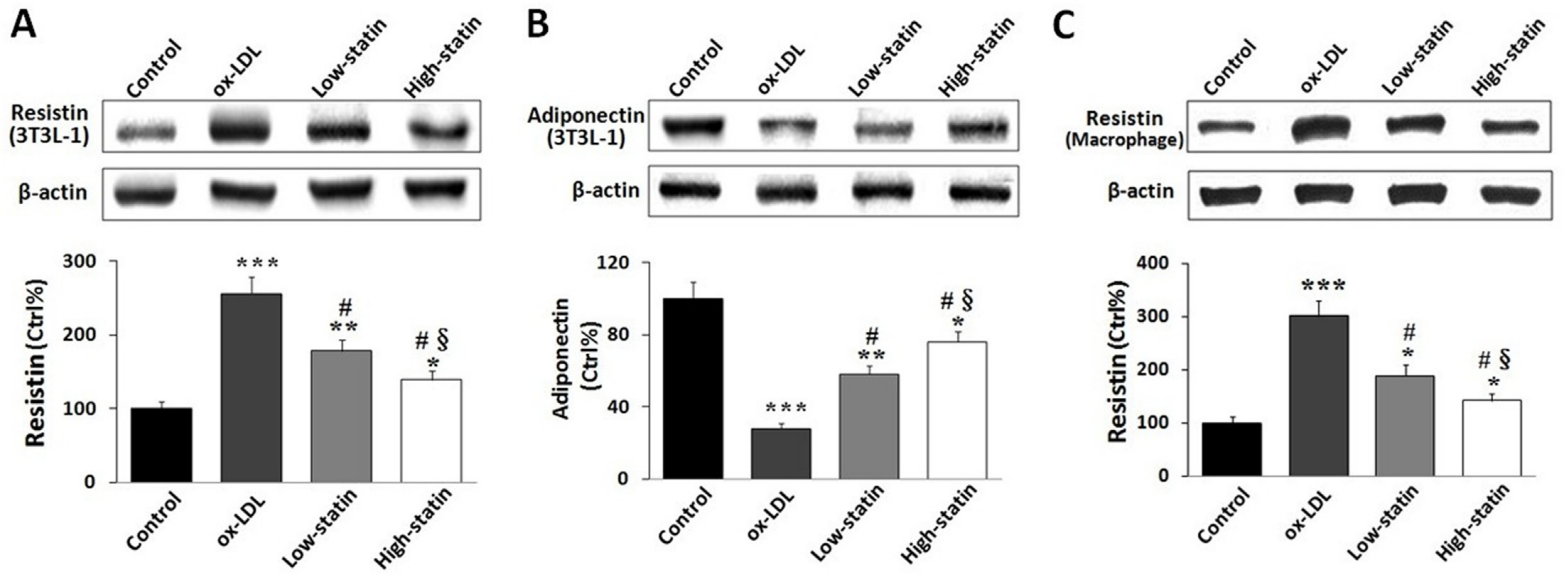
Effects of atorvastatin on resistin and adiponectin in cells. (A) Treatment with ox-LDL increased resistin expressions in mouse 3T3-L1 adipocytes. However, atorvastatin dose- dependently attenuated ox-LDL-induced elevation of resistin in adipocytes. (B) Ox-LDL treatment remarkably reduced adipocyte adiponectin expressions, but addition of atorvastatin dose-dependently ameliorated ox-LDL-induced reduction of adiponectin. (C) Treated with ox-LDL, resistin expressions were significantly increased in human macrophages. However, administration of atorvastatin dose-dependently reversed ox-LDL-induced up-regulation of resistin. *P < 0.05, **P < 0.01, ***P < 0.001 vs. Control; # P < 0.01 vs. ox-LDL group; § P < 0.05 vs. low-statin group.

Effects of atorvastatin on cellular expressions of resistin (in mouse 3T3-L1 adipocytes and human macrophages) and adiponectin (in mouse 3T3-L1 adipocytes) were also identified in this study. As shown in Fig 2, ox-LDL significantly increased resistin expressions in mouse 3T3-L1 adipocytes. However, addition of atorvastatin dose-dependently decreased the expressions of resistin in adipocytes treated with ox-LDL (Fig 2A). Conversely, ox-LDL remarkably down-regulated adiponectin expressions in 3T3-L1 adipocytes. When co-treated with atorvastatin, however, ox-LDL-induced down-regulation of adiponectin was dose-dependently affected in 3T3-L1 adipocytes (Fig 2B). Besides, similar changes of resistin expressions were observed in human macrophages as those in mouse 3T3-L1 adipocytes. In human macrophages, we demonstrated that ox-LDL treatment significantly up-regulated resistin expressions. However, co-treatment of atorvastatin dose-dependently resulted in reduction of macrophage resistin expressions (Fig 2C).

## Discussion

This study investigated the role of acute inflammation in adipokine imbalance during ACS. As stated above, ACS, as a group of acute ischemic attacks for human body, is likely to trigger an acute systemic inflammation as a defensive response for this ischemic tissue injury. In order to identify the ACS-activated inflammation, hs-CRP was used as an inflammatory predictor considering hs-CRP as a well-documented atherosclerosis-associated inflammatory biomarker [8]. In this study, we found higher hs-CRP levels in all CHD participants but more hs-CRP elevation was observed in ACS patients than SA patients. Two possible reasons could be explainable for higher hs-CRP levels in ACS patients. Firstly, since that inflammation levels are positively associated with atherosclerotic severity [9], more clinical severity could partly contribute to higher inflammation levels in ACS patients than SA patients. Secondly, the attack of ACS activates acute inflammation as a defensive response that would enhance inflammation levels in ACS patients. By contrast, unlike vulnerable plaque in ACS patients, stable plaque in SA patients immunes them from the rupture of plaque and then the attack of ACS [14]. Thus, SA patients have a lower probability of inflammation activation induced by acute ischemic cardiac events. In addition, our animal findings that our non-atherosclerotic AMI mice were witnessed higher hs-CRP levels implicate the role of AMI-related ischemic attack rather than atherosclerosis in the activation of inflammation in these mice. Considering the fact that our AMI animal models were established by surgical ligation of left coronary artery that means that the type of AMI in our animals should be categorized as a secondary AMI rather than an atherosclerosis-originated primary AMI [15], it supports our aforementioned viewpoint that the attack of ACS *per se* could lead to the activation of inflammation.

Just as two sides of the same coin, inflammation activation as an adaptive response is necessary for the tissue repair by ACS attack. However, it is likely that inflammation activation could predispose to adipokine imbalance [2,3]. The similar phenomenon has been observed in patients with heart failure [16,17]. For preserving the reduced systolic function of the heart, the renin–angiotensin–aldosterone system (RAAS) is activated during the early period of heart failure. However, persistent RAAS activation accelerates left ventricular remodeling and eventually decompensated heart failure [16,17]. Likewise, it is believed that inflammation activation during ACS has the potential to disturb the metabolism of adipokines. In fact, adipokine imbalance has been demonstrated in CHD patients [4,14]. Similarly, we also identified the presence of adipokine imbalance in all CHD subjects, but more severe adipokine imbalance (characterized with higher resistin and lower adiponectin levels) was observed in ACS patients than SA patients. Obviously, more severe atherosclerotic cardiac ischemia should be a contributor for more severity of adipokine imbalance in ACS patients. However, inflammation activated by ACS attack were enlisted as a candidate stimulator to confer worse adipokine imbalance in ACS patients. This speculation can be testified by our animal findings that AMI mice, rather than non-AMI mice, experienced adipokine imbalance characterized with higher circulating levels and adipose expressions of resistin and otherwise for adiponectin. Considering non-atherosclerotic AMI (*i*.*e*. the aforementioned secondary AMI [15]) in these mice, it implicates that adipokine imbalance in AMI mice is derived from the attack of AMI *per se*.

Furthermore, the role of inflammation in bridging ACS attack and adipokine imbalance had been investigated. Our correlation analysis demonstrated the consistent relationship between hs-CRP levels and the two adipokine levels (positive with resistin versus negative with adiponectin) in human and animals. Besides, we found that ox-LDL increased resistin and decreased adiponectin expressions *in vitro*. Accumulated evidences have established the crosstalk between chronic inflammation and adipocytokine metabolism during the development of atherosclerosis [5,6,15]. However, the worsened adipokine imbalance in our ACS subjects should not be entirely attributed to atherosclerosis-associated chronic inflammation. Instead, acute inflammation activated by ACS attack could be another important mechanism, which is also testified by our non-atherosclerotic AMI animal findings.

In this study, we also investigated the effects of statin anti-inflammation on AMI-induced adipokine imbalance. Previous studies by others have been demonstrated that statins can increase circulating adiponectin levels [18–20]. Similarly, we also found that atorvastatin has the potential to increase circulating concentrations and protein expressions of adiponectin *in vivo* and *in vitro*. However, the direct metabolic effects on adiponectin should not entirely responsible for the up-regulation of adiponectin, and the anti-inflammatory property of this agent could be another contributor. Anti-inflammatory property of statins has been well-established [7–9]. Unlike the timing of statin administration in human, atorvastatin was pre-treated for 3 weeks before AMI attack in our animals. This statin pre-treatment strategy was to investigate the short-term (at 24 hours after AMI) effects of statins on AMI-induced adipokine imbalance. Another consideration for statin pre-treatment is that the AMI in these mice was derived from non-atherosclerosis that is different from most naturally-occurred atherosclerotic AMI in humans [15]. So our animal findings were expected to identify the potential effects of atorvastatin on inflammation activation and then adipokine imbalance induced by the attack of AMI *per se* instead of atherosclerosis. As a result, our observations have shown that atorvastatin can effectively ameliorated adipokine imbalance in AMI mice. Besides, our study *in vitro* also demonstrated that statin attenuated resistin over-expression and adiponectin down-expression induced by ox-LDL. All together, statin anti-inflammation is believed to improve ACS attack-induced adipokine imbalance.

Since that inflammation is implicated as a bridging factor between ACS attack and adipokine imbalance in this study, another clinical setting likely predisposing to adipokine imbalance should be considered. Because that ischemia/reperfusion injury represents an acute attack [21,22], it is likely that inflammation could be activated in CHD patients undergoing percutaneous coronary intervention (PCI) or coronary artery bypass grafting (CABG) [19–21], which would lead to adipokine imbalance by the two revascularization procedures. And if so, statin pre-treatment before PCI/CABG is believed to reduce the incidence of adipokine imbalance. A recent meta-analysis shows that statin pre-treatment before PCI effectively improved periprocedural myocardial injury. Notably, the periprocedural benefits of statins were related with the baseline hs-CRP levels in patients, which displayed that the higher baseline hs-CRP levels, the greater benefits of statins. Obviously, the periprocedural benefits of statins should not contribute to the lipid-lowering effects of statins, because all trials included in this meta-analysis used a short-term pre-treatment with high-dose statin (median 0.5 days) that would not produce a significant lipid-lowering effect. Instead, anti-inflammatory property of statins was believed for the early cardioprotective benefits [22]. Together with our findings, the improvement of adipokine imbalance by repressing inflammation activation would provide another candidate explanation for the periprocedural benefits of statins in patients undergoing PCI or CABG. Nevertheless, the long-term outcomes of this statin pre-treatment strategy remain to be identified in future studies. Anyway, our study provide a novel insight into the use of statin pre-treatment before PCI/CABG procedures and the crosstalk between inflammation activation and adipokine imbalance would be a potential pharmacological target in ACS patients.
